# VEGF attenuates development from cardiac hypertrophy to heart failure after aortic stenosis through mitochondrial mediated apoptosis and cardiomyocyte proliferation

**DOI:** 10.1186/1749-8090-6-54

**Published:** 2011-04-16

**Authors:** Xiao H Xu, Jing Xu, Lei Xue, Hai L Cao, Xiang Liu, Yi J Chen

**Affiliations:** 1 Department of Thoracic and Cardiovascular Surgery, The First Affiliated Hospital of Nanjing Medical University, Nanjing, P. R. China

## Abstract

**Background:**

Aortic stenosis (AS) affects 3 percent of persons older than 65 years and leads to greater morbidity and mortality than other cardiac valve diseases. Surgery with aortic valve replacement (AVR) for severe symptomatic AS is currently the only treatment option. Unfortunately, in patients with poor ventricular function, the mortality and long-term outcome is unsatisfied, and only a minority of these patients could bear surgery. Our previous studies demonstrated that vascular endothelial growth factor (VEGF) protects cardiac function in myocardial infarction model through classic VEGF-PI3k-Akt and unclear mitochondrial anti-apoptosis pathways; promoting cardiomyocyte (CM) proliferation as well. The present study was designed to test whether pre-operative treatment with VEGF improves AS-induced cardiac dysfunction, to be better suitable for AVR, and its potential mechanism.

**Methods:**

Adult male mice were subjected to AS or sham operation. Two weeks later, adenoviral VEGF (Ad-VEGF), enhanced green fluorescence protein (Ad-EGFP, as a parallel control) or saline was injected into left ventricle free wall. Two weeks after delivery, all mice were measured by echocardiography and harvested for further detection.

**Results:**

AS for four weeks caused cardiac hypertrophy and left ventricular dysfunction. VEGF treatment increased capillary density, protected mitochondrial function, reduced CMs apoptosis, promoted CMs proliferation and eventually preserved cardiac function.

**Conclusions:**

Our findings indicate that VEGF could repair AS-induced transition from compensatory cardiac hypertrophy to heart failure.

## Background

Aortic stenosis (AS) is the most common cardiac valve disease, affecting about 3 percent of persons older than 65 years. Although the survival rate in asymptomatic patients is comparable to that in age- and sex-matched control patients, the average overall survival rate in symptomatic patients is 2-3 years [1]. For patients with severe symptomatic AS, surgical intervention with aortic valve replacement (AVR) is the only effective treatment available. Surgical mortality for isolated AVR in those with normal left ventricular function should be less than 1%. Successful valve replacement results in marked symptom relief and age-corrected survival becomes nearly normal. Yet, patients with AS and depressed ventricular function present high operative mortality and poor long-term outcome [2].

As the aortic valve area becomes smaller, the increased afterload on the left ventricle (LV) results in compensatory hypertrophy, which enables it to maintain systolic function. However, with time and sustained severe pressure overload, the LV dilates with impairment of contractile state and subsequent dysfunction. Although the molecular mechanisms involved in the transition from compensated hypertrophy to heart failure are poorly understood, the fundamental hypothesis is that, according to the nature of signaling stimulus, the cardiomyocytes (CMs) can either survive, leading to beneficial hypertrophy, or undergo apoptosis (programmed cell death), which promotes LV failure and dilation [3].

Vascular endothelial growth factor (VEGF) is an endothelial cell mitogen which has been recognized to have both angiogenenic and nonangiogenic role for cardiovascular system. VEGF regulates multiple cellular stress responses, including survival, proliferation, migration, and differentiation. Our previous studies have shown that VEGF could facilitate CMs regeneration and protect it from apoptosis which was related with the activation of phosphatidylinositol-3 kinase (PI-3K) and the upregulation of Bcl-2 expression [4]. Recently, Izumiya et al illustrated that sequestration of endogenous VEGF impairs adaptive cardiac hypertrophy through markedly reduced capillary density, increased myocardial fibrosis and upregulated collagen gene [5]. Moreover, Zisa et al found that intramuscular injection of recombinant human VEGF stimulates CMs regeneration, production of growth factors, and mobilization of progenitor cells, culminating in attenuation of disease progression and robust repair of the failing heart [6].

Take advantage these features of VEGF, we hypothesized that pre-operative VEGF treatment could improves the AS patients' condition, especially those with severe cardiac hypertrophy, to avoid the worsening of LV function and better suitable for AVR. Thus, it is important to test this hypothesis in an animal model of AS, and the results may be useful in designing and justifying future clinical trials.

## Methods

### Animals

The experiment protocols were approved by Animal Care and Use Committee of Nanjing Medical University. Ten-week-old male C57BL/6 mice were obtained from the Experimental Animal Center of Nanjing University (Nanjing, China). Animals were fed *ad libitum *standard mouse food pellets and tap water, and housed in groups of four to five mice with 12:12 hour light-dark cycles.

### Adenoviral-mediated Gene Transfer

Recombinant human adenoviral vectors are the most efficient gene delivery vehicles currently used for gene transfer in preclinical gene therapy models and in clinical cardiovascular gene therapy protocols because of the ease of their production and the broad cell tropism, particularly within the cardiovascular system which makes them widely used in myocardial gene therapy. All major cardiac cell types can be efficiently transduced by adenoviral vectors, both in vitro and in vivo. With regard to CMs, efficient in vivo transduction has been demonstrated in gene therapy models from several mammalian species [7]. In our study adenovirus vectors encoding VEGF (Ad-VEGF) and control adenovirus vectors encoding enhanced green fluorescence protein (Ad-EGFP) fragment were described previously [4]. We injected 1 × 10^8 ^plaque-forming units of Ad-VEGF or Ad-EGFP into left ventricle free wall two weeks after aorta ligature.

### Design of the study

Mice were randomly subjected to either aorta ligature-induced AS (n = 40) or sham operation (n = 10). Surgical mortality rates were 20% or 0%, respectively, for AS or sham operations. Two weeks after thoracic aortic constriction (TAC) (compensatory hypertrophy phase in this model), AS animals underwent midline sternotomy and further assigned to three groups: i) saline injected hypertrophied hearts (TAC group), ii) Ad-EGFP injected hypertrophied hearts (EGFP group), iii) Ad-VEGF injected hypertrophied hearts (VEGF group). Sham group mice also did midline sternotomy but no injection. Two weeks after viral delivery, all mice were examined by echocardiography and killed. The heart wet weight to body weight ratio and to tibia length ratio were calculated. Heart samples were frozen in liquid nitrogen and then stored at -80°C until analysis. Additional heart samples were used for electron microscopy and histological evaluation.

### Aortic stenosis model (aorta ligature)

Aortic stenosis was created by aorta ligature in accordance with method of transverse aortic constriction [8]. In brief, mice were anesthetized (with a mixture of 8 mg/100 g ketamine, 2mg/100 g xylazine, 0.6 mg/100 g atropine, and the pain reliever temgesic at 0.1 mg/100 g), intubated, and ventilated. Under a surgical microscope, a midline incision was made at the upper sternum. The aorta was dissected between the right innominate and the left carotid arteries and narrowed to a lumen size of 0.4 mm. Sham mice underwent similar surgery except for the narrowing of the aorta.

### Echocardiography

Two weeks after aorta ligature and viral delivery, the mice were undergone cardiac function assessment by transthoracic echocardiography with 12-MHz phased-array transducer (Hewlett Packard). The heart was imaged in the cross-sectional mode in parasternal long- and short-axis views of the LV. Average interventricular septum diameter (IVSd), LV posterior wall thickness (LVPW), LV ejection fraction (LVEF) and LV fractional shortening (FS) were measured from three consecutive cardiac cycles. All measurements were done by two experienced echocardiographer who were blinded to treatment assignment.

### Cardiac Hypertrophy

Tissue samples were taken from left ventricles, fixed with 4% formalin, embedded in paraffin, and cut into 3 μm thickness. Hematoxylin-eosin (HE) staining were performed using serial sections. CM cross-sectional area was measured by tracing the outlines of 100-200 CMs with a clear nucleus image per each heart using hematoxylin-eosin stained sections.

### Microvessel Density

For measurement of capillary density, sections taken perpendicular to the long axis of the LV were immunohistochemically stained with a specific primary antibody against von Willebrand Factor (vWF) (1:100, abcom). Capillary density was defined as the capillary to cardiomyocyte ratio.

### Cardiomyocyte Proliferation

To detect whether VEGF promotes cardiomyocyte proliferation, immunohistochemical analysis was performed for Ki-67 (1:100, Zymed Laboratories). Only nuclei that were clearly located in cardiomyocytes were counted.

### TUNNEL Assay

Apoptosis was determined by terminal deoxynucleotidyl transferase dUTP nick-end labeling (TUNEL) assay using a POD TUNEL kit (Roche, Mannheim, Germany). Apoptotic nuclei were identified manually to determine that only apoptotic cardiomyocyte nuclei were included. The number of TUNEL-positive cells was expressed as a percentage of total cells.

### Western Analysis

The LV tissue was homogenized with lysis buffer (pH 7.4) containing 25 mM Tris, 150 mM NaCl, 5 mM EDTA, 10 mM sodium pyrophosphate, 10 mM b-glycerophosphate, 1 mM sodium orthovanadate (Na_3_VO_4_), 1% (vol/vol) Triton X-100, 10% (vol/vol) glycerol, 1 mM dithiothreitol, 1 mM PMSF, and a protease inhibitor cocktail (Sigma, St. Louis, MO). The total protein homogenate (20-50 μg) was separated by SDS-PAGE and transferred onto PVDF membranes. The expression levels of important signaling molecules, VEGF, and apoptosis-related proteins were detected using antibodies against OPA1, Bax, Bcl-xL, Akt and p-Akt from Cell Signaling Technology.

### Electron Microscopy

Standard transmission electron microscopy (EM) was performed as previously described [9]. Digital images of sequential fields were collected for analysis. To determine the population and size of the mitochondria, the EM images were analyzed with Photoshop CS3, using the counting and area analysis function, in an approach similar to that reported by other investigators.

### Statistical analysis

Data were analyzed using SPSS software package (Version 14.0; SPSS Inc, Cary, NC, USA) and are reported as mean ± standard error of the mean. One-way ANOVA was used for comparison among and between groups, or Kruskal-Wallis test if normality was not passed, followed by Bonferroni or Dunn post-hoc analysis when appropriate. Values of P < 0.05 were considered statistically significant.

## Results

### Cardiac Function and Morphology

**Figure 1:**
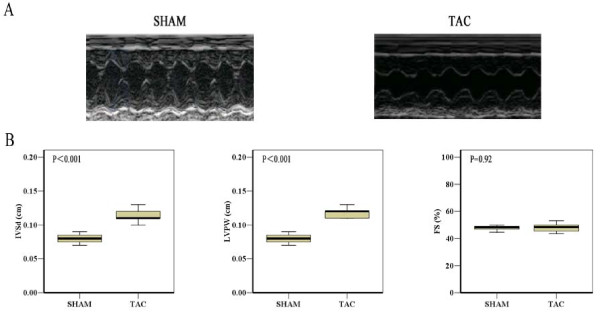
**Two weeks aortic ligature results in cardiac hypertrophy and normal cardiac function**. **A**, Representative transthoracic M-mode echocardiogram for sham and TAC mice **B**, IVSd, LVPW, and LVFS data between sham and TAC mice.

**Figure 2:**
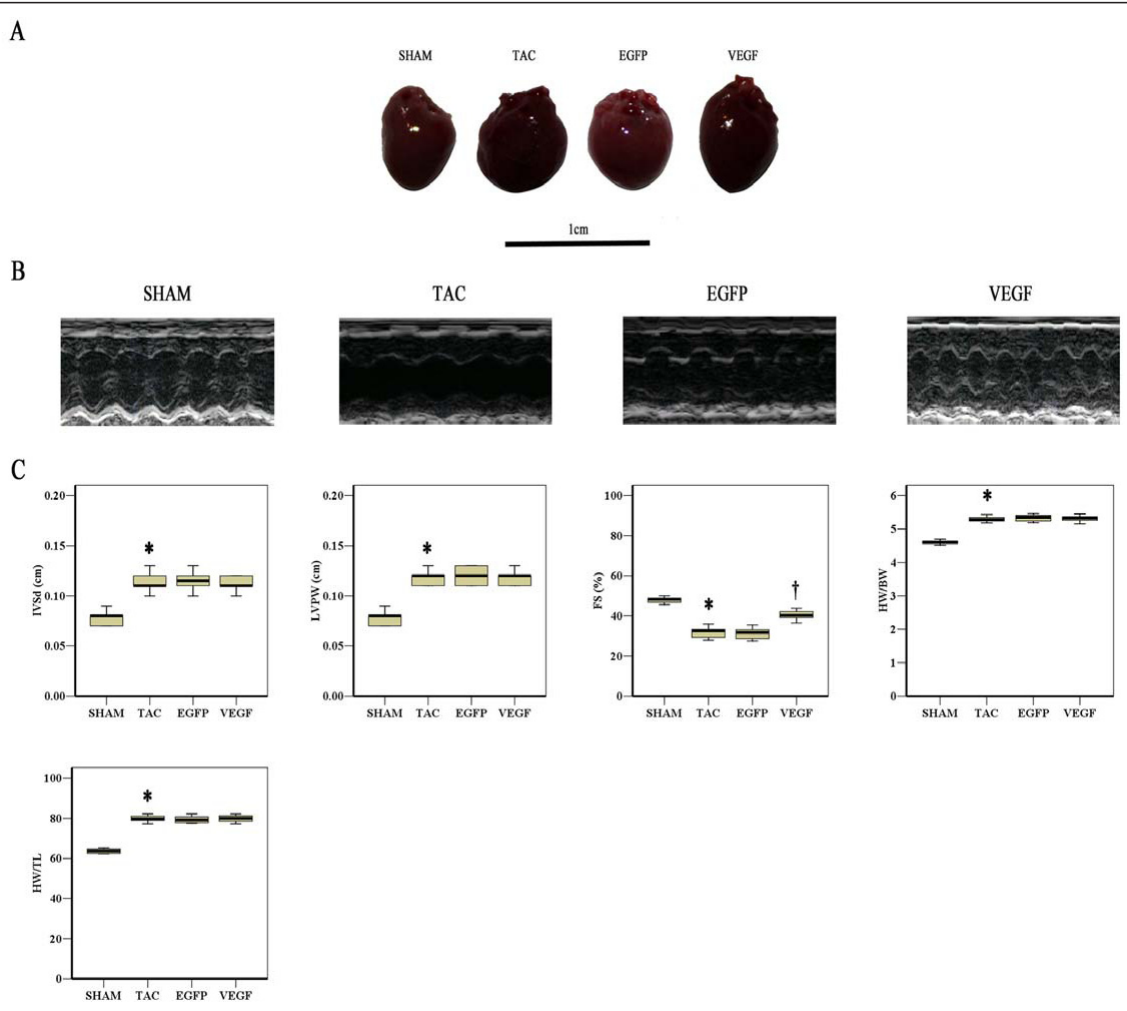
**Ad-VEGF treatment improves heart function at two weeks after viral delivery**. **A**, Representative photographs of the hearts for sham, TAC, EGFP and VEGF mice **B**, Representative transthoracic M-mode echocardiogram for sham, TAC, EGFP and VEGF mice **C**, IVSd, LVPW, LVFS, ratios of heart weight to body weight and heart weight to tibia length data for sham, TAC, EGFP and VEGF mice. *P < 0.05, TAC vs. Sham; †*P *< 0.05, VEGF vs. TAC.

**Figure 3:**
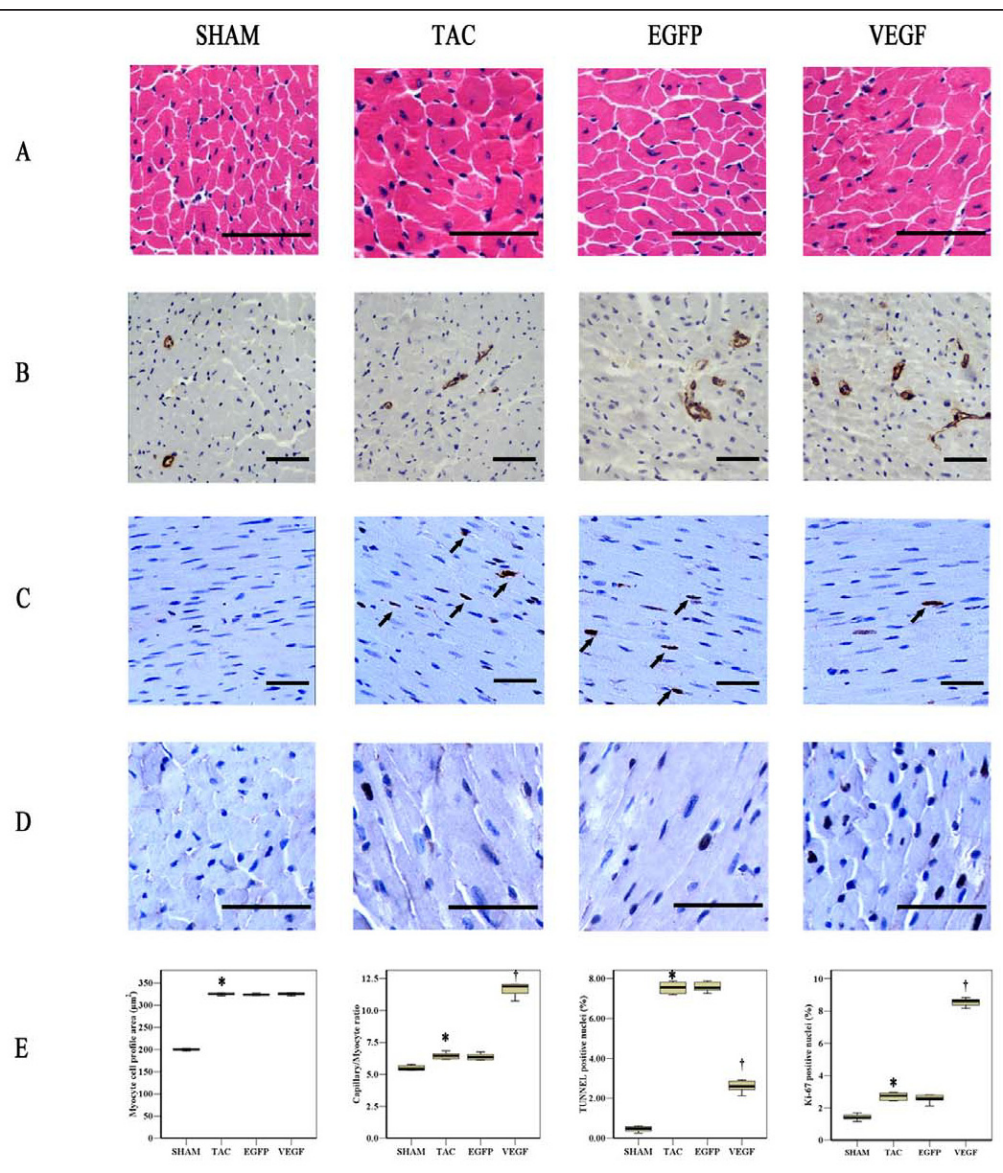
**VEGF overexpression exerts increased capillary density, reduced CMs apoptosis, promoted CMs proliferation two weeks after viral injection**. **A**, Representative histological micrographs of the LV myocardium stained with hematoxylin-eosin **B**, vWF immunostaining to identify capillary density **C**, Histological identification of apoptotic CMs for TUNEL **D**, Ki-67 staining to detect CMs proliferation **E**, Quantitative analysis of the cross-sectional area of CMs, and ratios of capillary, TUNEL positive nuclei and Ki-67 positive nuclei to myocyte in LV myocardium (Bars = 100 μm). *P < 0.05, TAC vs. Sham; †*P *< 0.05, VEGF vs. TAC.

Two weeks after aorta ligature, AS mice displayed the increased LV posterior wall thickness (LVPW), interventricular septal thickness (IVSd) (*P *< 0.001) and similar LV fractional shortening (LVFS), LV ejection fraction (LVEF) (*P *= 0.92) compared with sham operated mice, indicating compensatory cardiac hypertrophy and normal systolic function (Figure 1). However at two weeks after adenoviral injection, TAC mice showed a marked LV enlargement and signs of diminished cardiac function - i.e., reduced LVEF and LVFS (*P *< 0.01). Treatment with Ad-VEGF prevented the reductions of LVEF and LVFS (*P *< 0.05), with no significant difference in LVPW, IVSd, heart weight/body weight ratio and heart weight/tibia length ratio, as compared to TAC animals (Figure 2). Histological evaluation further confirmed that the cross-sectional area of CMs increased in theses three AS groups compared to sham, although VEGF treatment had no effect on CMs hypertrophy (Figure 3A, E).

### Microvessel Density

Figure 3B shows representative photomicrographs of the four different groups. Capillary density was significantly increased in TAC compared to sham group (*P *< 0.05). VEGF treatment revealed an augmentation of neovascularization after induction of AS. In the VEGF group we observed a 45% increase in capillary density relative to TAC group (*P *< 0.01). The differences between TAC and EGFP group were not statistically significant.

### Cardiomyocyte Apoptosis

The number of TUNEL positive cells was significantly higher in mice with AS, compared to sham (*P *< 0.05, Figure 3C). Treatment with VEGF resulted in a 66% reduction apoptotic CMs (*P *< 0.01). However, the numbers of TUNEL positive cells did not differ significantly between the TAC and EGFP groups.

### Cardiomyocyte Proliferation

Immunostaining for the cell proliferation marker Ki-67 was used to confirm VEGF-induced CMs regeneration. Induction of AS resulted in a 2 fold increased in the expression of Ki-67 in the TAC group (Figure 3D). VEGF significantly promoted CMs proliferation by approximately increased 3 fold as many Ki67 positive nuclei as TAC group (*P *< 0.01). Additionally no significant difference was found between TAC and EGFP group.

### Mitochondrial Morphology and Function

**Figure 4:**
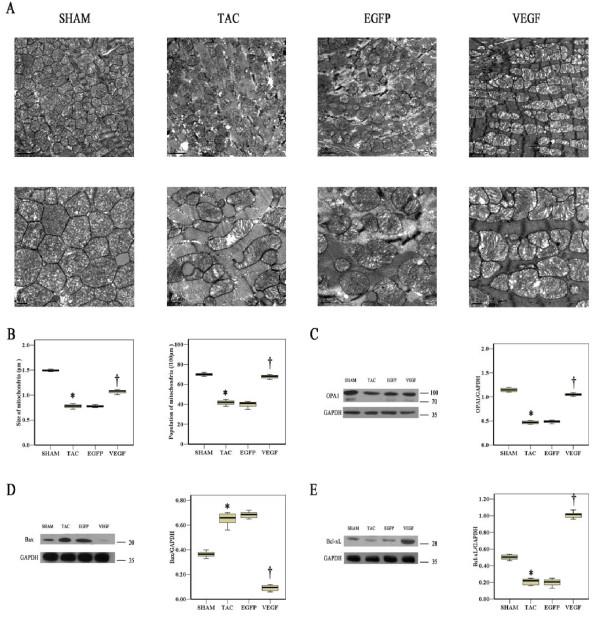
**VEGF injection maintains mitochondrial fission and fusion balance and protects mitochondrial regulated apoptosis two weeks after viral injection**. **A**, Representative EM for sham, TAC, EGFP and VEGF mice heart **B**, Graphs summarize the mitochondria per area and average mitochondrial size **C**, **D**, **E**, Representative Western blots and the results of quantitative analysis for OPA1, Bax and BcL-xL protein expression. *P < 0.05, TAC vs. Sham; †*P *< 0.05, VEGF vs. TAC.

EM was used to analyze mitochondrial fission and fusion changes in AS induced heart failure. The mitochondria in the TAC mice heart were disorganized and smaller (Figure 4A). The absolute number of mitochondria per area was significantly increased and the individual mitochondrial cross-sectional areas were significantly decreased as compared to sham group. VEGF injection significant decreased the absolute number of mitochondria per area and increased the individual mitochondrial cross-sectional areas (P < 0.05, Figure 4B). Expression of OPA1, a mitochondrial fusion protein, was decreased in TAC group, as observed by western blotting, which would be seen with a loss of the fusion/fission balance. VEGF improved the reduction of OPA1 expression (Figure 4C, P < 0.05), this suggests an important role for OPA1 in the progressive deterioration of the failing heart. Meanwhile the mitochondrial apoptosis pathway regulation proteins Bcl-xL and Bax were also detected by western blotting. The up-regulation of Bcl-xL and down-regulation of Bax expression further substantiated the anti-apoptosis effect of VEGF (Figure 4D, E, P < 0.05).

### In Vivo VEGF and Phosphorylated Akt Expression

**Figure 5:**
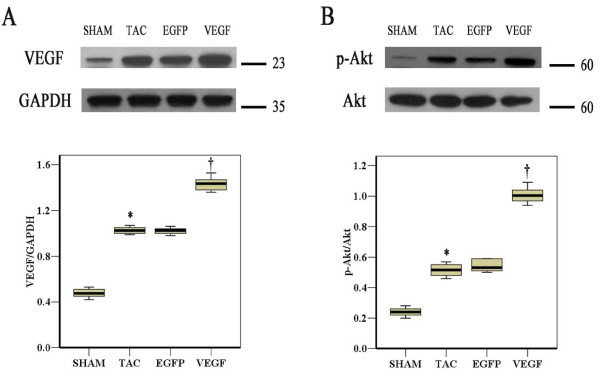
**Ad-VEGF increases expression of VEGF (**A**), p-Akt (**B**) protein two weeks after viral injection**. *P < 0.05, TAC vs. Sham; †*P *< 0.05, VEGF vs. TAC.

After 2 weeks injection of adenovirus containing VEGF in hypertrophied hearts, western blot analysis showed that levels of VEGF were increased in VEGF group compared with that in other groups (Fig 5A, *P *< 0.05). To investigate whether VEGF could activate PI3K-Akt signaling in the myocardium, we examined the level of phospho-Akt and Akt in the myocardium. As shown in Figure 5B, significant increase in the ratio of phosphor-Akt/Akt was observed in the VEGF group compared with that in other groups (*P *< 0.05). Thus, Ad-VEGF injection increased expression of VEGF and phospho-Akt in myocardium.

## Discussion

AS-induced LV hypertrophy is an initially expected response in order for the CMs to generate additional force to overcome the increase in pressure load. The initial response may become decompensated when CMs degenerate as a result of inflammatory response, oxidative stress, apoptosis, fibrosis and progressive LV dilation with a progressive decline in cardiac pump function. In the present study, we found that two weeks AS mice presented cardiac hypertrophy and normal cardiac function. Whereas, at 4 weeks after aorta ligature, remarkable pulmonary congestion and LV dysfunction were exhibited in the TAC mice, underwent a transition from compensatory cardiac hypertrophy to heart failure. VEGF injection in compensatory hypertrophy heart was found to attenuate LV remodeling and to improve cardiac function through increased capillary density, preserved mitochondrial function, promoted CMs proliferation, as well as reduced CMs apoptosis.

It is well known that myocardial apoptosis has been shown to be a critical determinant of unfavorable LV remodeling and play an important role in the progression of AS. However the underlying mechanisms by which the heart loses CMs in heart failure are not completely understood [3].

One important component of the myocardial remodeling process is neoangiogenesis. After AS, neoangiogenesis is normally unable to compensate for the blood supply and to support the tissue growth required for contractile compensation and the greater demands of the hypertrophied myocardium; this may contribute to the death of myocardium, leading to progressive CMs apoptosis and secondary fibrosis replacement. Our study suggested an increase in angiogenesis in VEGF animals, and this may contribute importantly to the reduction in remodeling and apoptosis. Ferrata [10] demonstrated that VEGF could control the recruitment of endothelial progenitor cells and facilitate the proliferation of endothelial cells. Additionally Alon et al. [11] found that VEGF as a mitogen for vascular endothelial cells is crucial for vascular development and endothelial cell survival. In our model, therefore, we would predict that Ad-VEGF injection may enhance LV local VEGF expression, protect endothelial cells, promote endothelial progenitor cell recruitment, and improve vascular development, resulting in enhanced angiogenesis.

Mitochondria also have a critical role in regulating apoptosis. CMs function at a high metabolic state, requiring large amounts of high-energy phosphates, and as a consequence, mitochondria are abundant in these cells [12,13]. Mitochondrial fission and fusion, described recently and most extensively in dilated cardiomyopathy and ischaemic cardiomyopathy, occur constantly and are thought to be critical for normal mitochondrial function [14]. If fission is interrupted, large networks of fused mitochondria occur. If fusion fails, become small and fragmented. Abnormalities in fission and fusion can lead to apoptosis, which is an important mechanism of CMs loss in heart failure [15-17]. OPA1 is a mitochondrial fusion protein which is important for maintaining normal cristae structure and function, for preserving the inner membrane structure and for protecting CMs from apoptosis. As shown in our study, 4 weeks after AS, the mitochondria of CMs become small and dysfunctional, this is similar with early studies on DCM and ICM. VEGF treatment maintained mitochondrial fission and fusion balance, increased the expression of inhibiting apoptosis proteins OPA1 and Bcl-xL, decreased the expression of promoting apoptosis protein Bax, leading to prevention mitochondrial apoptotic regulated pathways.

Phosphoinositide 3-kinase and its downstream target serine/threonine kinase Akt are also recognized as another most critical pathways in regulating CMs activation, inflammatory responses and apoptosis [18,19]. Activation of PI3K/Akt-dependent signaling has been shown to prevent CMs apoptosis and protect the myocardium [20]. Our results found that the level of phosphor-Akt was elevated and the apoptosis of myocardium was reduced in VEGF group compared with that in TAC group. So we demonstrated that the effect of VEGF was mediated partially through the PI3K/Akt-dependent anti-apoptotic mechanism.

In current, myocardial regeneration has become the hotspot and challenge of clinical treatment for heart failure. It may offer possibilities that could supplement apoptosis-conduced CMs shortage and maintain the absolute numbers of CMs, as well as improve cardiac function. Evidence is accumulating to suggest that VEGF exerts potent pleiotropic effects on the myocardium in the setting of acute myocardial infarction and chronic heart failure as well. In our previous study we demonstrated that overexpression VEGF could mobilize marrow stem cell and accelerate CMs regeneration in myocardial infarction model [21]. Furthermore Zisa et al. [6] shown that VEGF stimulated proliferation, migration, and growth factor production of CMs, which provides evidence for CMs regeneration and progenitor cell expansion. Our results indicate that a significant effect of VEGF injections can be attributed to enhanced CMs proliferation in the myocardium following AS. Consequently, we could forecast that VEGF may contribute to mobilize bone marrow stem cells and promote stem cells differentiation into CMs and endothelial cells. Differentiated CMs render it possible that reenter the cell cycle and recommence proliferation. Additionally VEGF may give rise to reduce apoptosis of viable CMs, thereby contributing to CMs proliferation. However, the precise mechanisms of VEGF-induced CMs proliferation need to be evaluated in further studies.

## Conclusions

In summary, the results of the present study using the mouse model of AS bear out the cardioprotective, angiogenic, proliferative, and anti-apoptotic effects of VEGF and its possible molecular mechanism. It is known that reduced LV ejection fraction and increased LV cavity size before AVR are associated with poor postoperative recovery. Although the correlation to human or clinical data remains to be proved, it is our anticipation that the number of AS patients who are suitable for AVR will be increased, the mortality following AVR will be decreased, and postoperative recovery will be improved if VEGF pre-operative treatment can be demonstrated to be effective in clinical trials.

## List of abbreviations

AS: Aortic stenosis; AVR: Aortic valve replacement; VEGF: Vascular endothelial growth factor; CM: Cardiomyocyte; Ad-VEGF: Adenoviral VEGF; Ad-EGFP: Adenoviral enhanced green fluorescence protein; LV: Left ventricle; PI-3K: Phosphatidylinositol-3 kinase; IVSd: Interventricular septum diameter; LVPW: LV posterior wall thickness; LVEF: LV ejection fraction; FS: LV fractional shortening; HE: Hematoxylin-eosin; vWF: Willebrand Factor; TUNEL: Terminal deoxynucleotidyl transferase dUTP nick-end labeling; EM: Electron microscopy.

## Competing interests

The authors declare that they have no competing interests.

## Authors' contributions

XHX, JX and YJC participated in the design of the study and coordination, LX and HLC participated in the data collect and modified the manuscript, XL performed the statistical analysis and helped to draft and modified the manuscript. All authors read and approved the final manuscript.
